# Supplementary figures and images for: Scutellarin combined with lidocaine exerts antineoplastic effect in human glioma associated with repression of epidermal growth factor receptor signaling
Source: PLoS One. 2025 Jan 31;20(1):e0318031. doi: 10.1371/journal.pone.0318031 (PMC11785270; doi:10.1371/journal.pone.0318031)

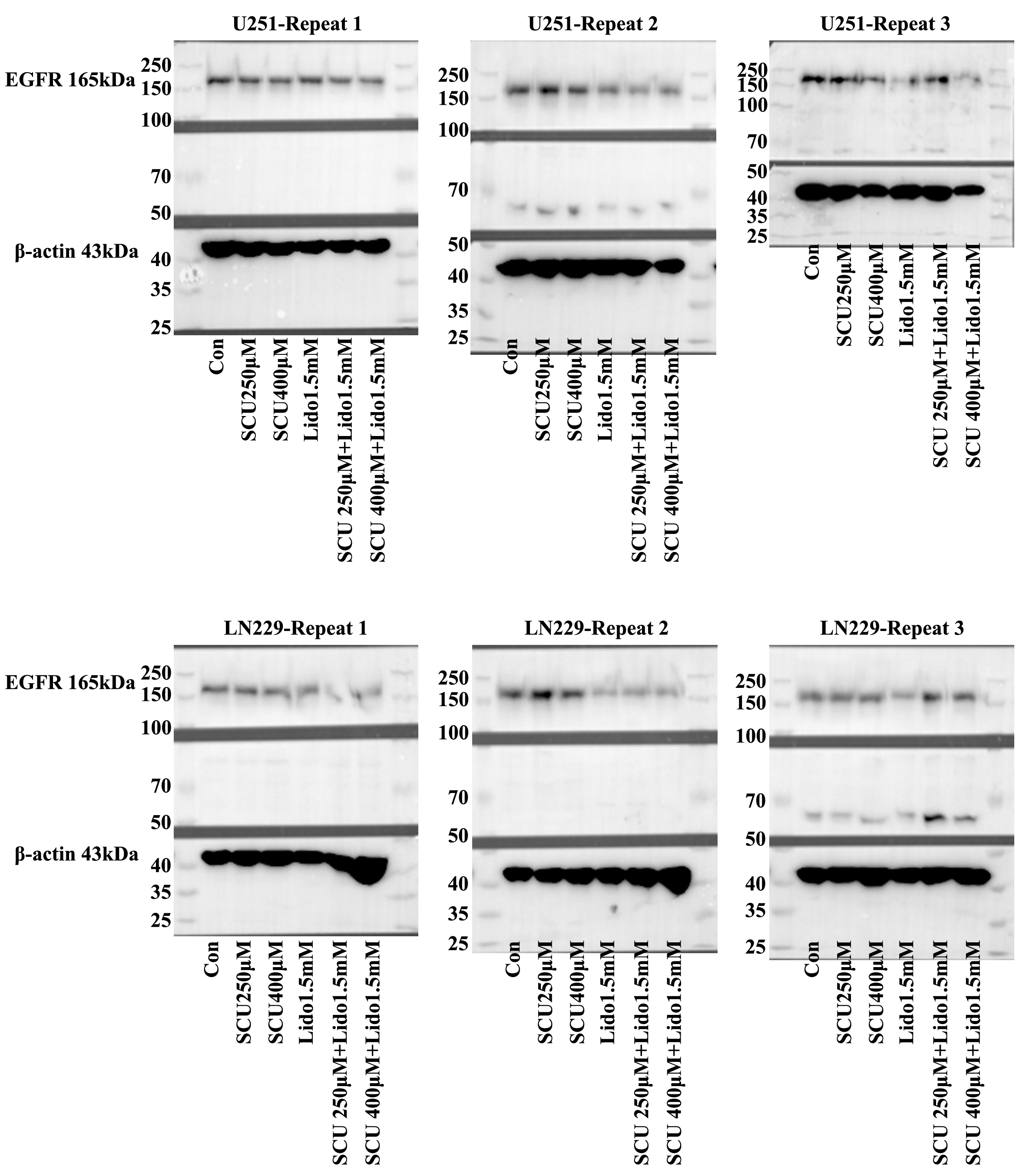

Supplement: S1 Fig — (TIF) [file pone.0318031.s001.tif]
