# Supplementary material for: Scutellarin combined with lidocaine exerts antineoplastic effect in human glioma associated with repression of epidermal growth factor receptor signaling
Source: PLoS One. 2025 Jan 31;20(1):e0318031. doi: 10.1371/journal.pone.0318031 (PMC11785270; doi:10.1371/journal.pone.0318031)
Supplement: S1 File — (PDF) [file pone.0318031.s017.pdf]

U251 报告

标本名：U251　　检验时间：2018/1/23 10:55

仪器：NovoCyte 451150212708　　软件：NovoExpress 1.2.5

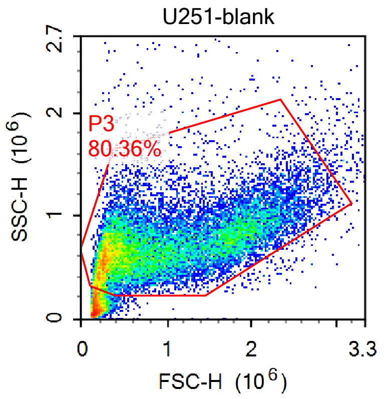

Gate	% All
All	100.00%
P3	80.36%

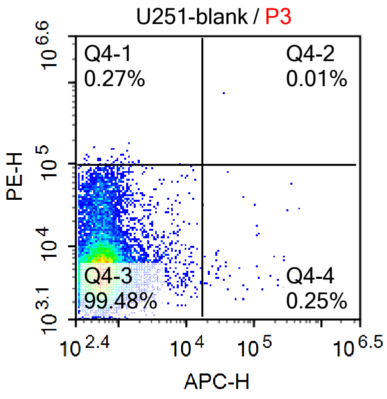

Gate	% P3	% All
P3	100.00%	80.36%
Q4-1	0.27%	0.21%
Q4-2	0.01%	0.00%
Q4-3	99.48%	79.94%

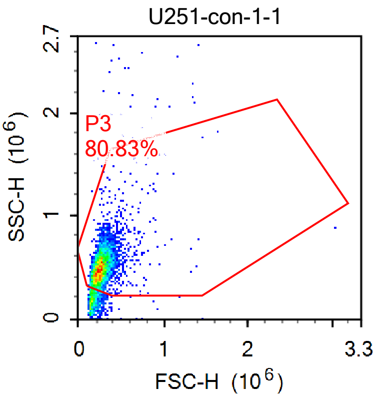

Gate	% All
All	100.00%
P3	80.83%

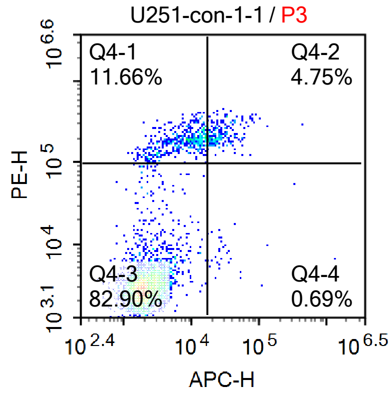

Gate	% P3	% All
P3	100.00%	80.83%
Q4-1	11.66%	9.42%
Q4-2	4.75%	3.84%
Q4-3	82.90%	67.00%
Q4-4	0.69%	0.56%

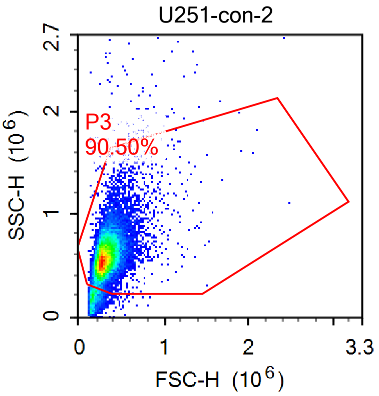

Gate	% All
All	100.00%
P3	90.50%

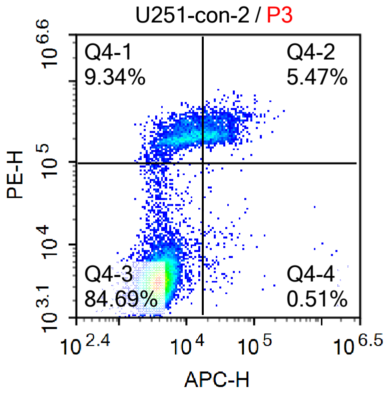

Gate	% P3	% All
P3	100.00%	90.50%
Q4-1	9.34%	8.45%
Q4-2	5.47%	4.95%
Q4-3	84.69%	76.65%
Q4-4	0.51%	0.46%

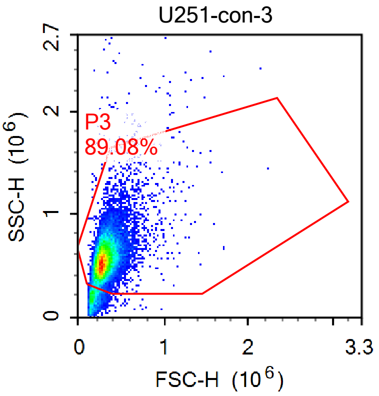

Gate	% All
All	100.00%
P3	89.08%

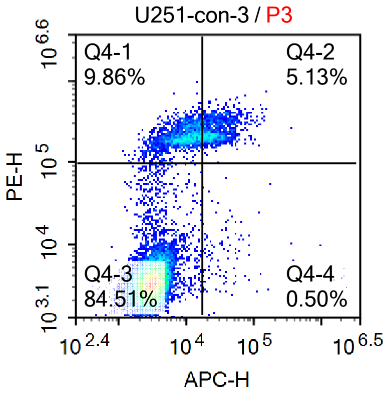

Gate	% P3	% All
P3	100.00%	89.08%
Q4-1	9.86%	8.79%
Q4-2	5.13%	4.57%
Q4-3	84.51%	75.28%

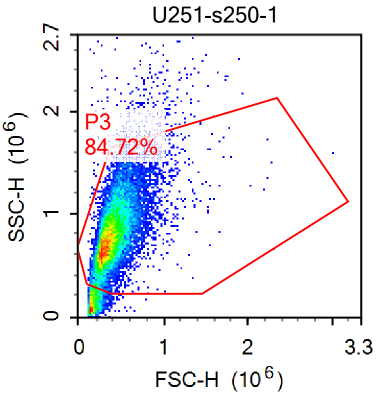

Gate	% All
All	100.00%
P3	84.72%

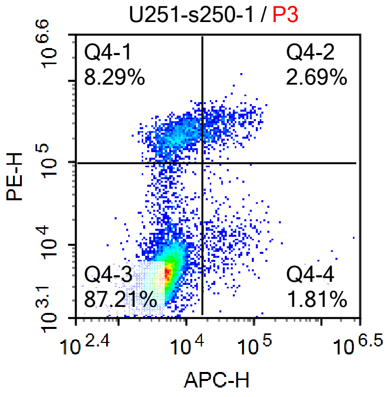

Gate	% P3	% All
P3	100.00%	84.72%
Q4-1	8.29%	7.02%
Q4-2	2.69%	2.28%
Q4-3	87.21%	73.89%
Q4-4	1.81%	1.53%

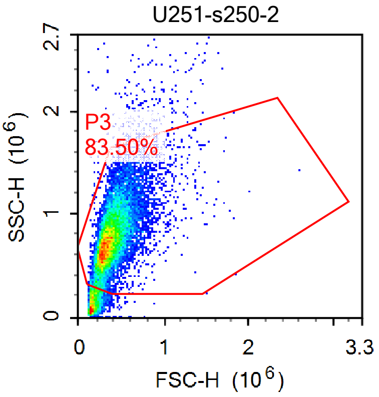

Gate	% All
All	100.00%
P3	83.50%

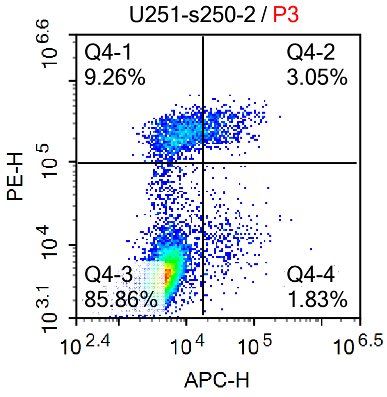

Gate	% P3	% All
P3	100.00%	83.50%
Q4-1	9.26%	7.73%
Q4-2	3.05%	2.54%
Q4-3	85.86%	71.69%
Q4-4	1.83%	1.53%

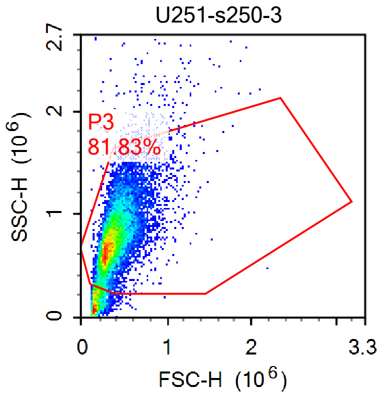

Gate	% All
All	100.00%
P3	81.83%

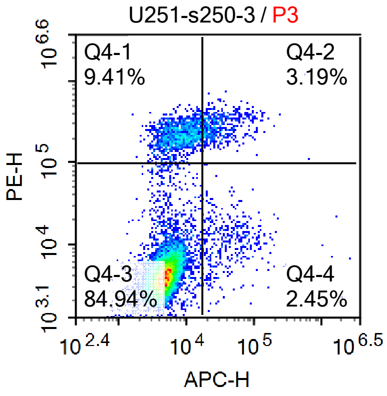

Gate	% P3	% All
P3	100.00%	81.83%
Q4-1	9.41%	7.70%
Q4-2	3.19%	2.61%
Q4-3	84.94%	69.50%

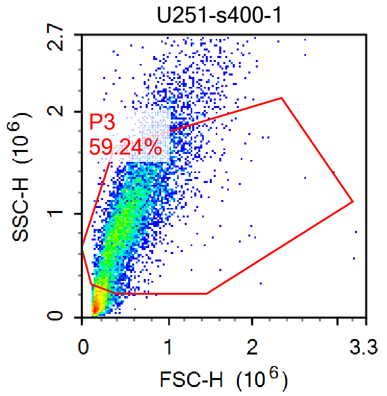

Gate	% All
All	100.00%
P3	59.24%

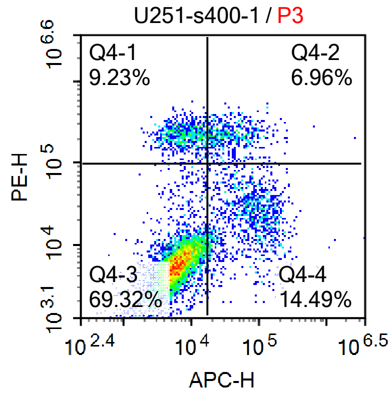

Gate	% P3	% All
P3	100.00%	59.24%
Q4-1	9.23%	5.47%
Q4-2	6.96%	4.12%
Q4-3	69.32%	41.07%
Q4-4	14.49%	8.58%

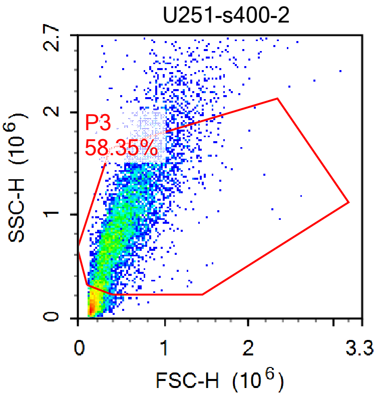

Gate	% All
All	100.00%
P3	58.35%

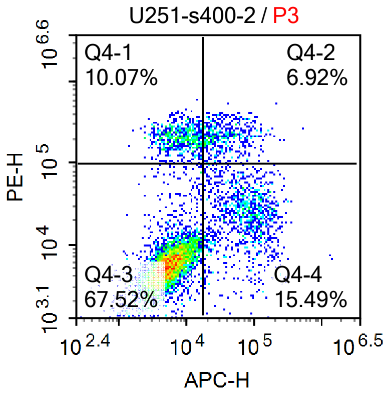

Gate	% P3	% All
P3	100.00%	58.35%
Q4-1	10.07%	5.87%
Q4-2	6.92%	4.04%
Q4-3	67.52%	39.40%
Q4-4	15.49%	9.04%

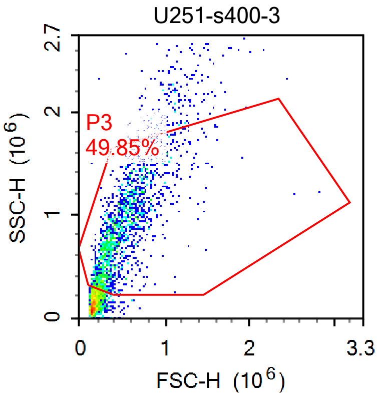

Gate	% All
All	100.00%
P3	49.85%

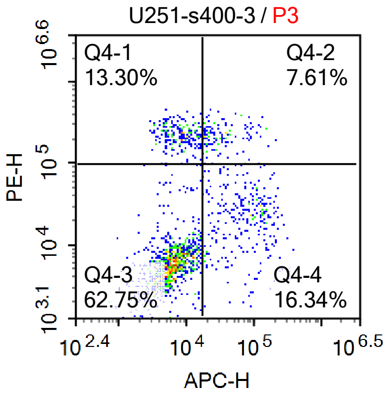

Gate	% P3	% All
P3	100.00%	49.85%
Q4-1	13.30%	6.63%
Q4-2	7.61%	3.79%
Q4-3	62.75%	31.28%

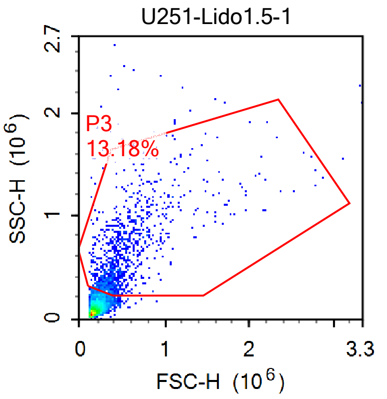

Gate	% All
All	100.00%
P3	13.18%

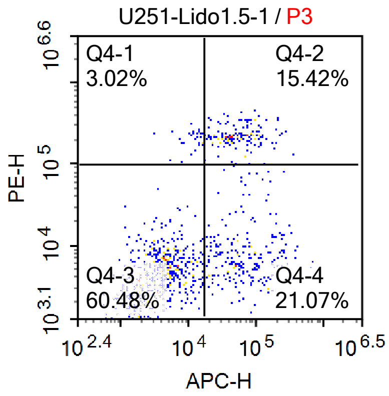

Gate	% P3	% All
P3	100.00%	13.18%
Q4-1	3.02%	0.40%
Q4-2	15.42%	2.03%
Q4-3	60.48%	7.97%
Q4-4	21.07%	2.78%

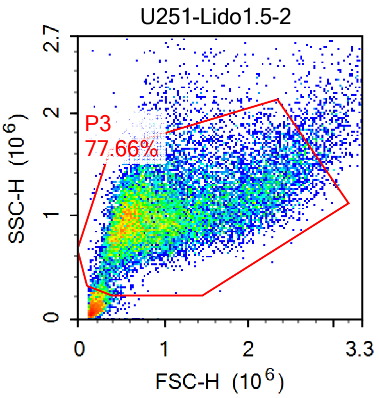

Gate	% All
All	100.00%
P3	77.66%

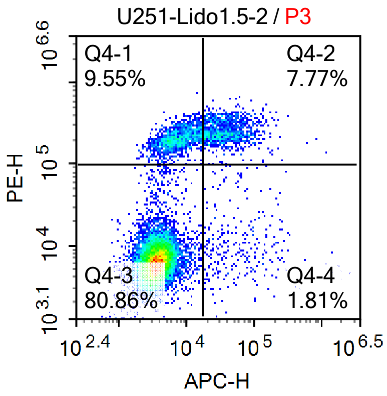

Gate	% P3	% All
P3	100.00%	77.66%
Q4-1	9.55%	7.42%
Q4-2	7.77%	6.04%
Q4-3	80.86%	62.79%
Q4-4	1.81%	1.41%

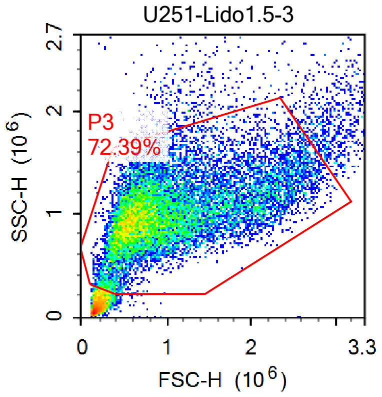

Gate	% All
All	100.00%
P3	72.39%

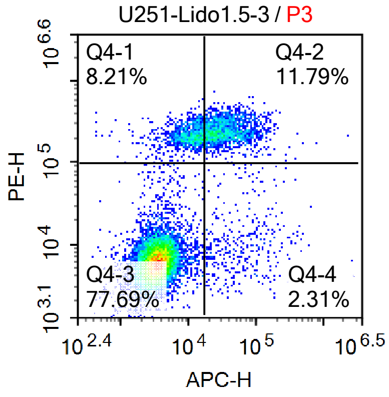

Gate	% P3	% All
P3	100.00%	72.39%
Q4-1	8.21%	5.94%
Q4-2	11.79%	8.53%
Q4-3	77.69%	56.24%

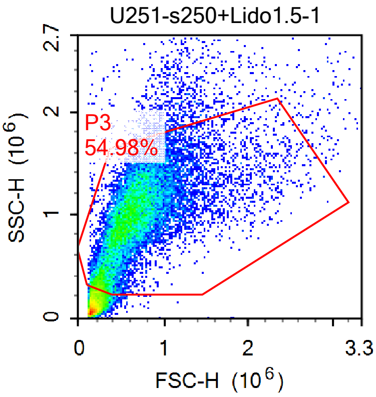

Gate	% All
All	100.00%
P3	54.98%

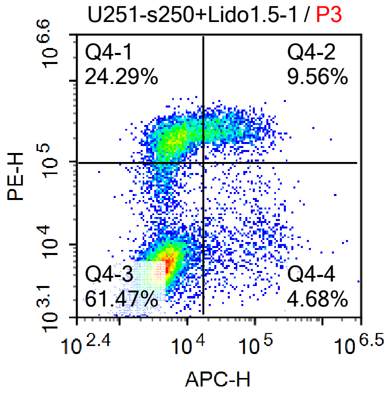

Gate	% P3	% All
P3	100.00%	54.98%
Q4-1	24.29%	13.36%
Q4-2	9.56%	5.26%
Q4-3	61.47%	33.80%
Q4-4	4.68%	2.57%

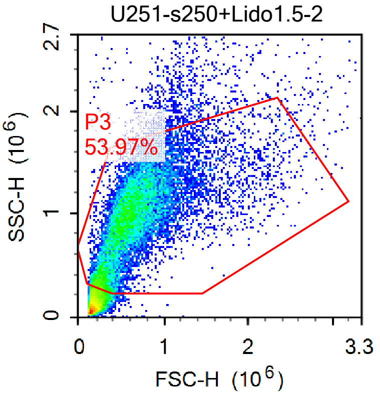

Gate	% All
All	100.00%
P3	53.97%

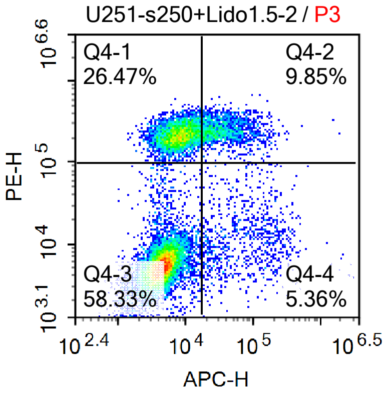

Gate	% P3	% All
P3	100.00%	53.97%
Q4-1	26.47%	14.28%
Q4-2	9.85%	5.31%
Q4-3	58.33%	31.48%
Q4-4	5.36%	2.89%

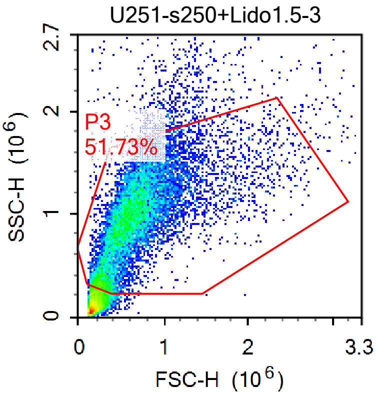

Gate	% All
All	100.00%
P3	51.73%

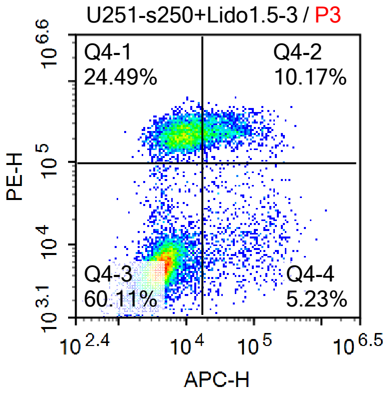

Gate	% P3	% All
P3	100.00%	51.73%
Q4-1	24.49%	12.67%
Q4-2	10.17%	5.26%
Q4-3	60.11%	31.09%

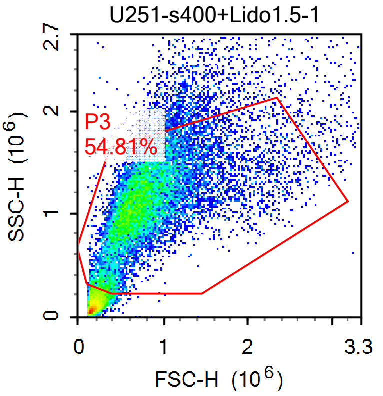

Gate	% All
All	100.00%
P3	54.81%

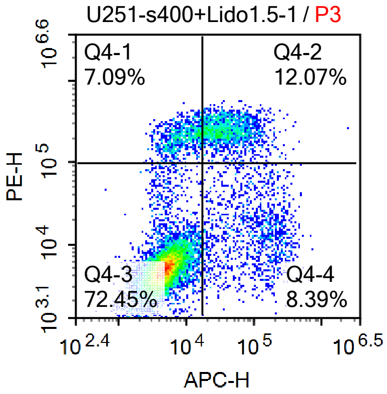

Gate	% P3	% All
P3	100.00%	54.81%
Q4-1	7.09%	3.89%
Q4-2	12.07%	6.61%
Q4-3	72.45%	39.71%
Q4-4	8.39%	4.60%

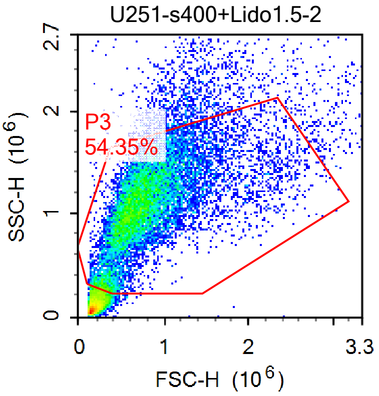

Gate	% All
All	100.00%
P3	54.35%

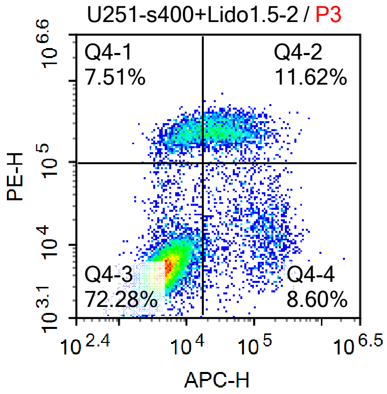

Gate	% P3	% All
P3	100.00%	54.35%
Q4-1	7.51%	4.08%
Q4-2	11.62%	6.31%
Q4-3	72.28%	39.28%
Q4-4	8.60%	4.67%

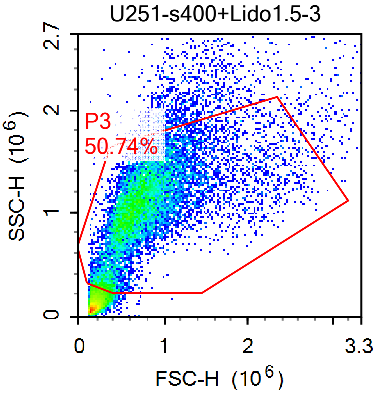

Gate	% All
All	100.00%
P3	50.74%

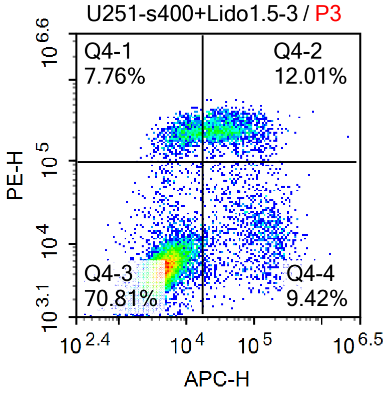

Gate	% P3	% All
P3	100.00%	50.74%
Q4-1	7.76%	3.94%
Q4-2	12.01%	6.10%
Q4-3	70.81%	35.93%
