# Supplementary material for: Scutellarin combined with lidocaine exerts antineoplastic effect in human glioma associated with repression of epidermal growth factor receptor signaling
Source: PLoS One. 2025 Jan 31;20(1):e0318031. doi: 10.1371/journal.pone.0318031 (PMC11785270; doi:10.1371/journal.pone.0318031)
Supplement: S2 File — (PDF) [file pone.0318031.s018.pdf]

标本1 报告

标本名：标本1　　检验时间：2018/1/21 20:45

仪器：NovoCyte 451150212708　　软件：NovoExpress 1.2.5

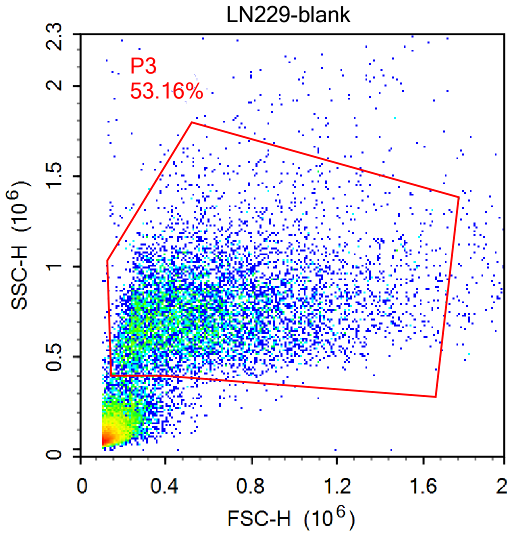

Gate	% All
All	100.00%
P3	53.16%

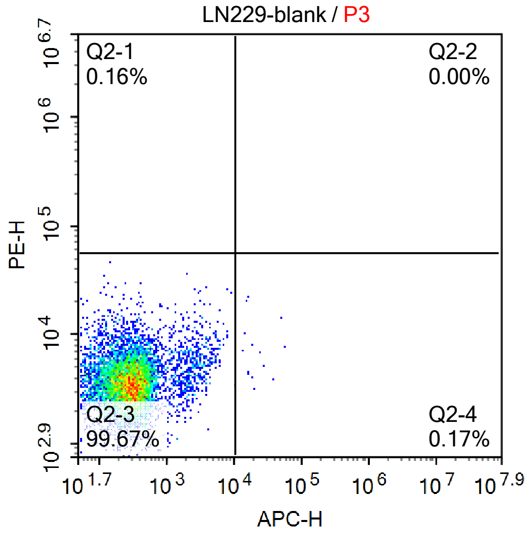

Gate	% P3	% All
P3	100.00%	53.16%
Q2-1	0.16%	0.09%
Q2-2	0.00%	0.00%
Q2-3	99.67%	52.99%
Q2-4	0.17%	0.09%

样本统计表格 - LN229-blank

Gate	% Parent	% Grandparent	% All
All			
P3	53.16%		53.16%
Q2-1	0.16%	0.09%	0.09%
Q2-2	0.00%	0.00%	0.00%
Q2-3	99.67%	52.99%	52.99%
Q2-4	0.17%	0.09%	0.09%

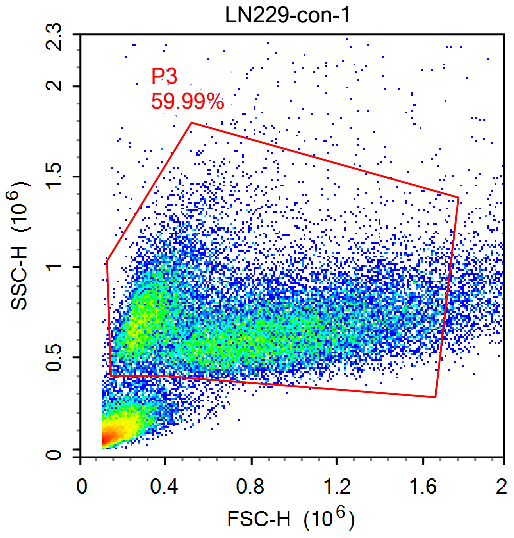

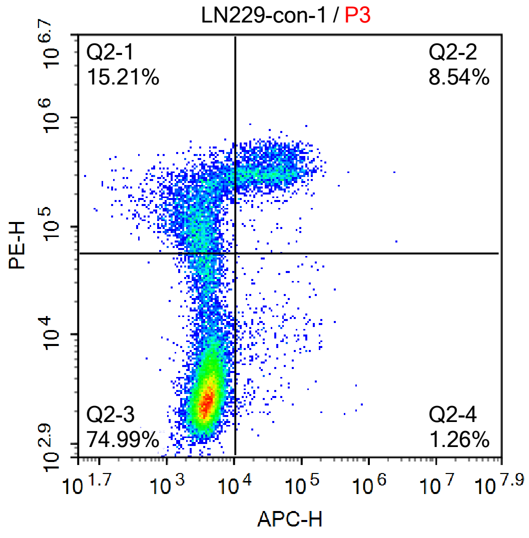

Gate	% All
All	100.00%
P3	59.99%

Gate	% P3	% All
P3	100.00%	59.99%
Q2-1	15.21%	9.12%
Q2-2	8.54%	5.12%
Q2-3	74.99%	44.99%
Q2-4	1.26%	0.75%

样本统计表格 - LN229-con-1

Gate	% Parent	% Grandparent	% All
All			
P3	59.99%		59.99%
Q2-1	15.21%	9.12%	9.12%
Q2-2	8.54%	5.12%	5.12%
Q2-3	74.99%	44.99%	44.99%
Q2-4	1.26%	0.75%	0.75%

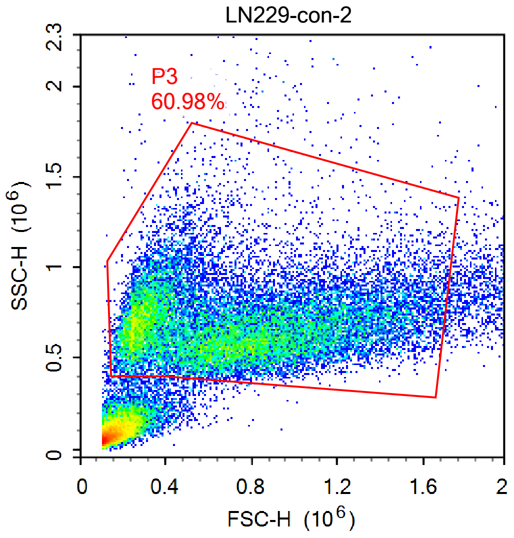

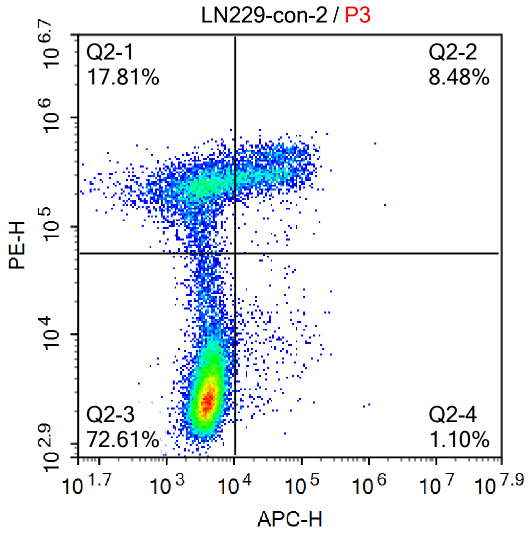

Gate	% All
All	100.00%
P3	60.98%

Gate	% P3	% All
P3	100.00%	60.98%
Q2-1	17.81%	10.86%
Q2-2	8.48%	5.17%
Q2-3	72.61%	44.28%
Q2-4	1.10%	0.67%

样本统计表格 - LN229-con-2

Gate	% Parent	% Grandparent	% All
All			
P3	60.98%		60.98%
Q2-1	17.81%	10.86%	10.86%
Q2-2	8.48%	5.17%	5.17%
Q2-3	72.61%	44.28%	44.28%
Q2-4	1.10%	0.67%	0.67%

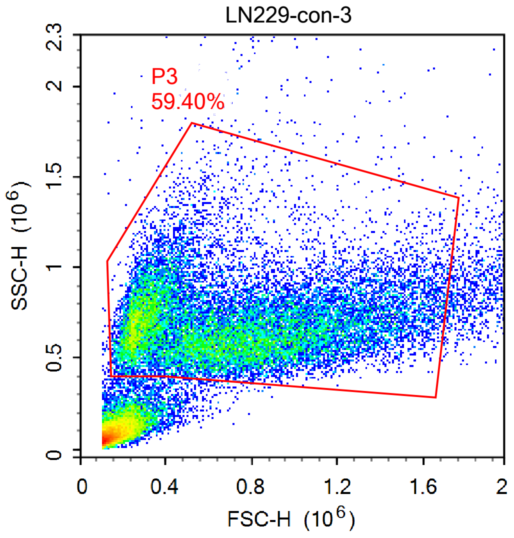

Gate	% All
All	100.00%
P3	59.40%

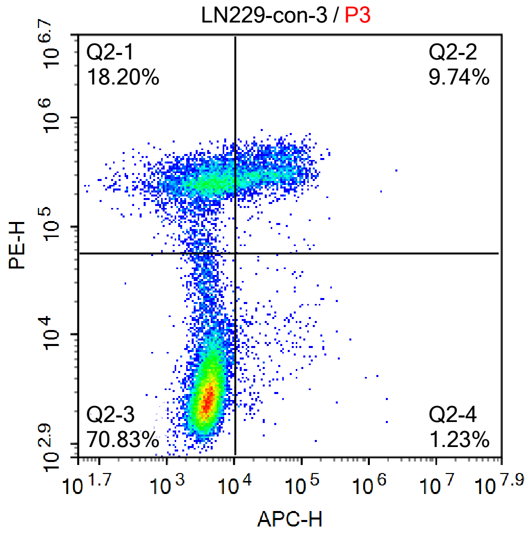

Gate	% P3	% All
P3	100.00%	59.40%
Q2-1	18.20%	10.81%
Q2-2	9.74%	5.78%
Q2-3	70.83%	42.07%
Q2-4	1.23%	0.73%

样本统计表格 - LN229-con-3

Gate	% Parent	% Grandparent	% All
All			
P3	59.40%		59.40%
Q2-1	18.20%	10.81%	10.81%
Q2-2	9.74%	5.78%	5.78%
Q2-3	70.83%	42.07%	42.07%
Q2-4	1.23%	0.73%	0.73%

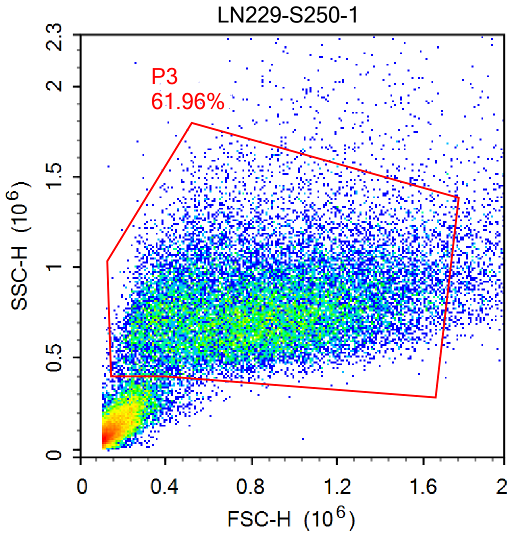

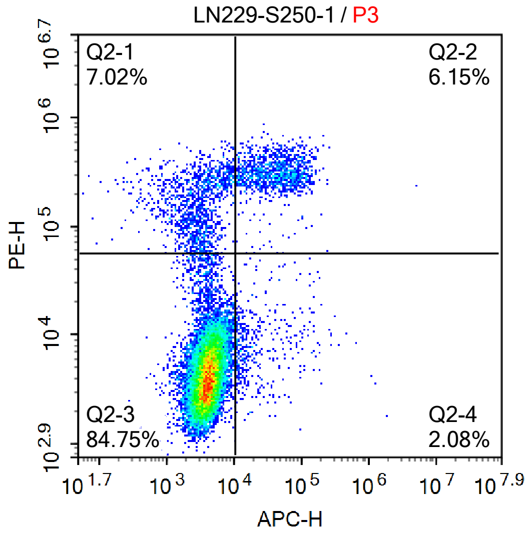

Gate	% All
All	100.00%
P3	61.96%

Gate	% P3	% All
P3	100.00%	61.96%
Q2-1	7.02%	4.35%
Q2-2	6.15%	3.81%
Q2-3	84.75%	52.51%
Q2-4	2.08%	1.29%

样本统计表格 - LN229-S250-1

Gate	% Parent	% Grandparent	% All
All			
P3	61.96%		61.96%
Q2-1	7.02%	4.35%	4.35%
Q2-2	6.15%	3.81%	3.81%
Q2-3	84.75%	52.51%	52.51%
Q2-4	2.08%	1.29%	1.29%

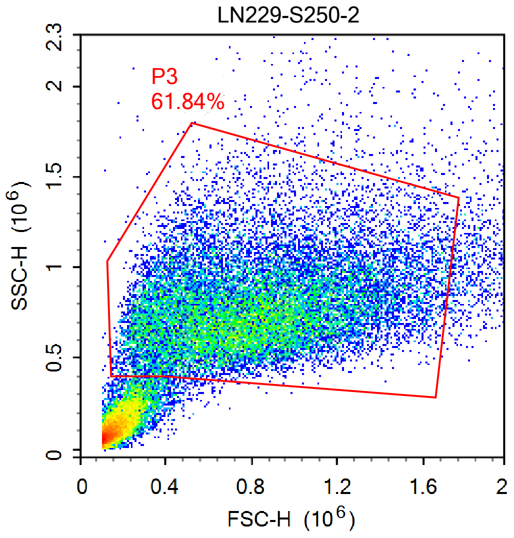

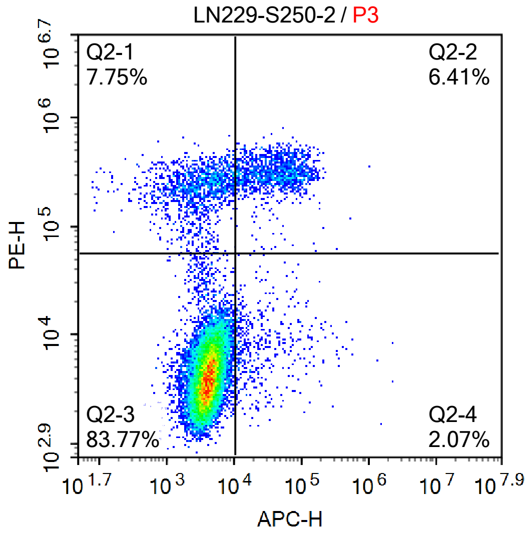

Gate	% All
All	100.00%
P3	61.84%

Gate	% P3	% All
P3	100.00%	61.84%
Q2-1	7.75%	4.79%
Q2-2	6.41%	3.96%
Q2-3	83.77%	51.81%
Q2-4	2.07%	1.28%

样本统计表格 - LN229-S250-2

Gate	% Parent	% Grandparent	% All
All			
P3	61.84%		61.84%
Q2-1	7.75%	4.79%	4.79%
Q2-2	6.41%	3.96%	3.96%
Q2-3	83.77%	51.81%	51.81%
Q2-4	2.07%	1.28%	1.28%

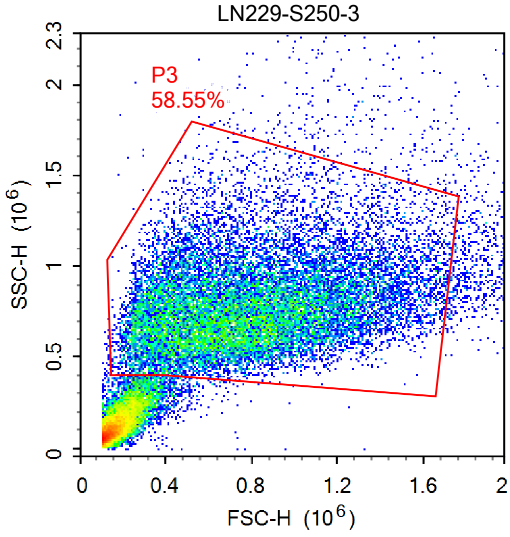

Gate	% All
All	100.00%
P3	58.55%

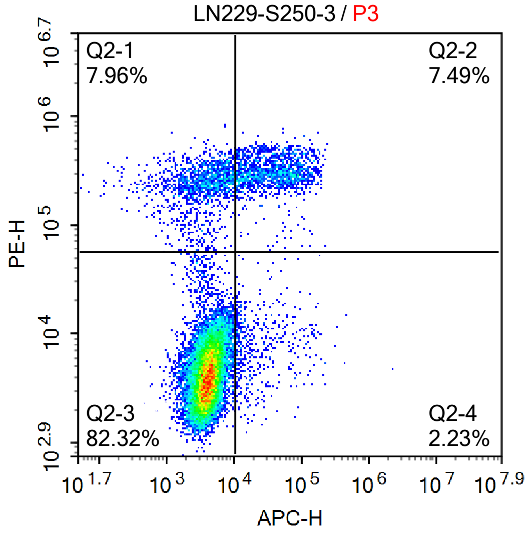

Gate	% P3	% All
P3	100.00%	58.55%
Q2-1	7.96%	4.66%
Q2-2	7.49%	4.38%
Q2-3	82.32%	48.20%
Q2-4	2.23%	1.31%

样本统计表格 - LN229-S250-3

Gate	% Parent	% Grandparent	% All
All			
P3	58.55%		58.55%
Q2-1	7.96%	4.66%	4.66%
Q2-2	7.49%	4.38%	4.38%
Q2-3	82.32%	48.20%	48.20%
Q2-4	2.23%	1.31%	1.31%

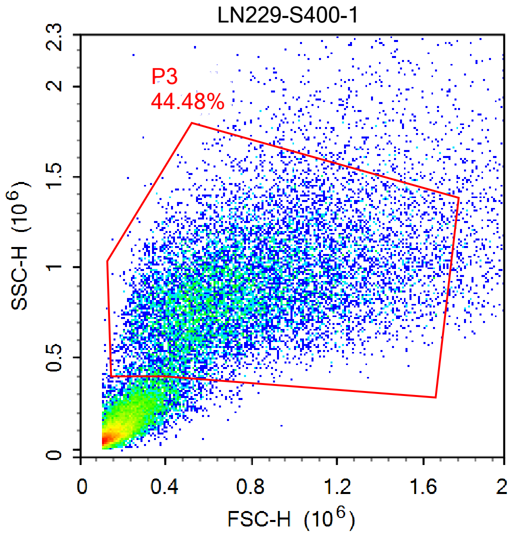

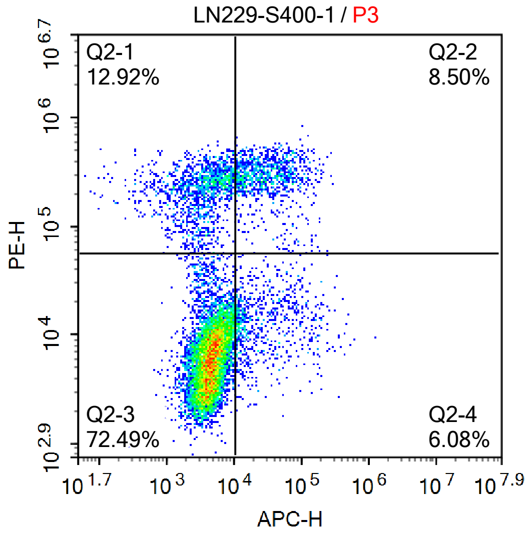

Gate	% All
All	100.00%
P3	44.48%

Gate	% P3	% All
P3	100.00%	44.48%
Q2-1	12.92%	5.75%
Q2-2	8.50%	3.78%
Q2-3	72.49%	32.24%
Q2-4	6.08%	2.71%

样本统计表格 - LN229-S400-1

Gate	% Parent	% Grandparent	% All
All			
P3	44.48%		44.48%
Q2-1	12.92%	5.75%	5.75%
Q2-2	8.50%	3.78%	3.78%
Q2-3	72.49%	32.24%	32.24%
Q2-4	6.08%	2.71%	2.71%

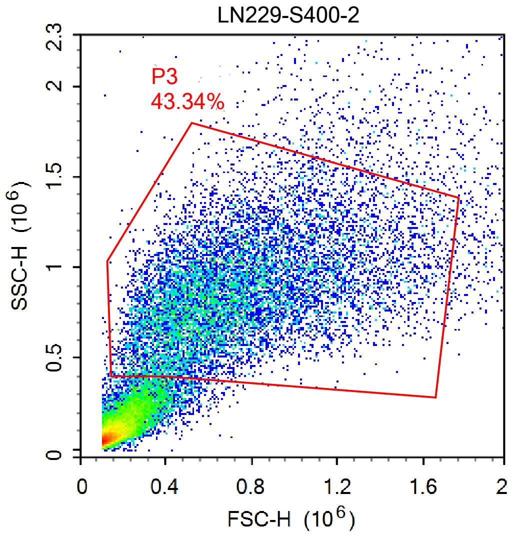

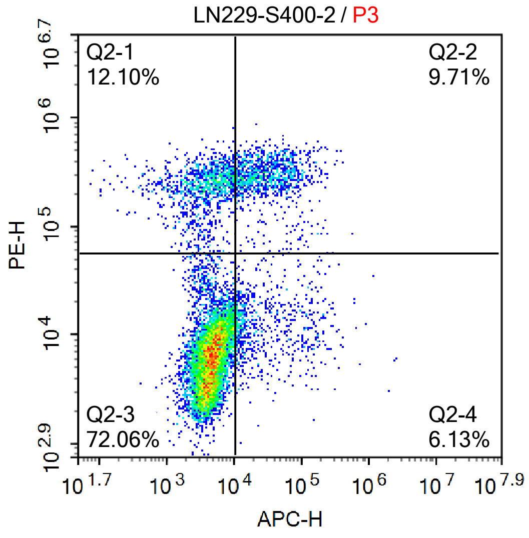

Gate	% All
All	100.00%
P3	43.34%

Gate	% P3	% All
P3	100.00%	43.34%
Q2-1	12.10%	5.25%
Q2-2	9.71%	4.21%
Q2-3	72.06%	31.23%
Q2-4	6.13%	2.66%

样本统计表格 - LN229-S400-2

Gate	% Parent	% Grandparent	% All
All			
P3	43.34%		43.34%
Q2-1	12.10%	5.25%	5.25%
Q2-2	9.71%	4.21%	4.21%
Q2-3	72.06%	31.23%	31.23%
Q2-4	6.13%	2.66%	2.66%

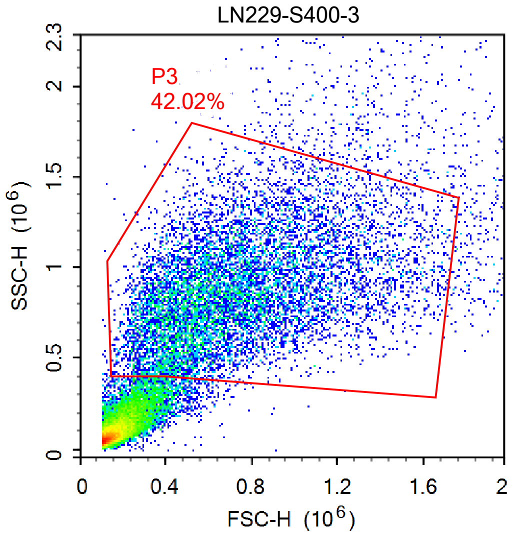

Gate	% All
All	100.00%
P3	42.02%

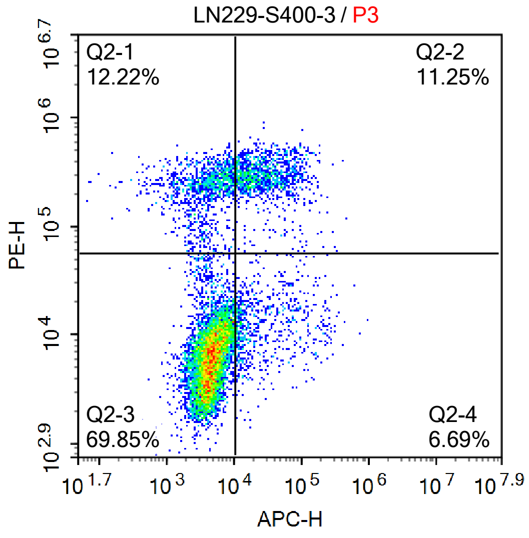

Gate	% P3	% All
P3	100.00%	42.02%
Q2-1	12.22%	5.13%
Q2-2	11.25%	4.73%
Q2-3	69.85%	29.35%
Q2-4	6.69%	2.81%

样本统计表格 - LN229-S400-3

Gate	% Parent	% Grandparent	% All
All			
P3	42.02%		42.02%
Q2-1	12.22%	5.13%	5.13%
Q2-2	11.25%	4.73%	4.73%
Q2-3	69.85%	29.35%	29.35%
Q2-4	6.69%	2.81%	2.81%

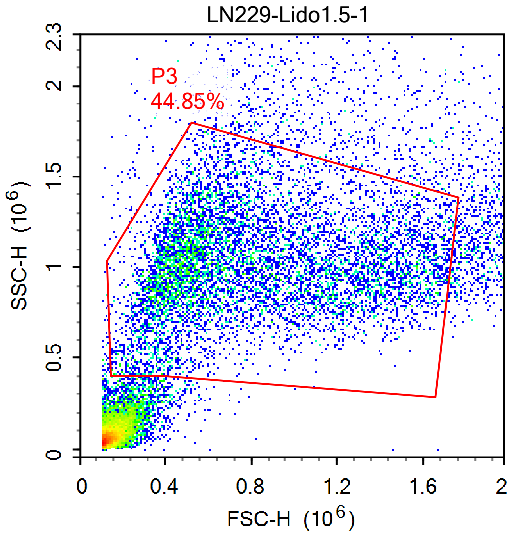

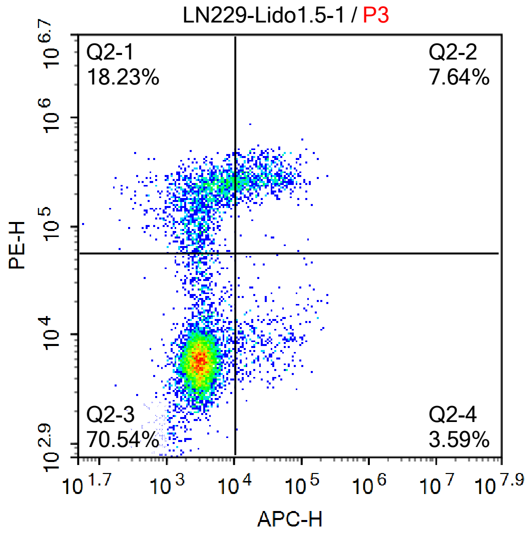

Gate	% All
All	100.00%
P3	44.85%

Gate	% P3	% All
P3	100.00%	44.85%
Q2-1	18.23%	8.18%
Q2-2	7.64%	3.43%
Q2-3	70.54%	31.64%
Q2-4	3.59%	1.61%

样本统计表格 - LN229-Lido1.5-1

Gate	% Parent	% Grandparent	% All
All			
P3	44.85%		44.85%
Q2-1	18.23%	8.18%	8.18%
Q2-2	7.64%	3.43%	3.43%
Q2-3	70.54%	31.64%	31.64%
Q2-4	3.59%	1.61%	1.61%

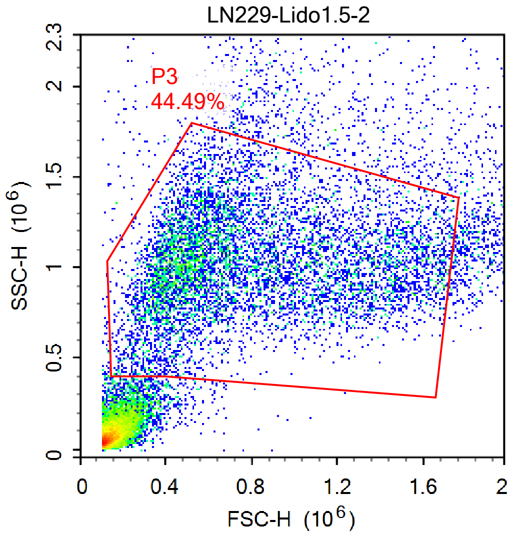

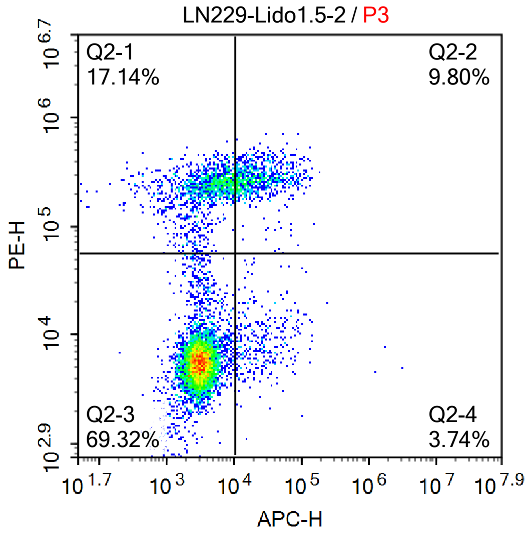

Gate	% All
All	100.00%
P3	44.49%

Gate	% P3	% All
P3	100.00%	44.49%
Q2-1	17.14%	7.63%
Q2-2	9.80%	4.36%
Q2-3	69.32%	30.84%
Q2-4	3.74%	1.66%

样本统计表格 - LN229-Lido1.5-2

Gate	% Parent	% Grandparent	% All
All			
P3	44.49%		44.49%
Q2-1	17.14%	7.63%	7.63%
Q2-2	9.80%	4.36%	4.36%
Q2-3	69.32%	30.84%	30.84%
Q2-4	3.74%	1.66%	1.66%

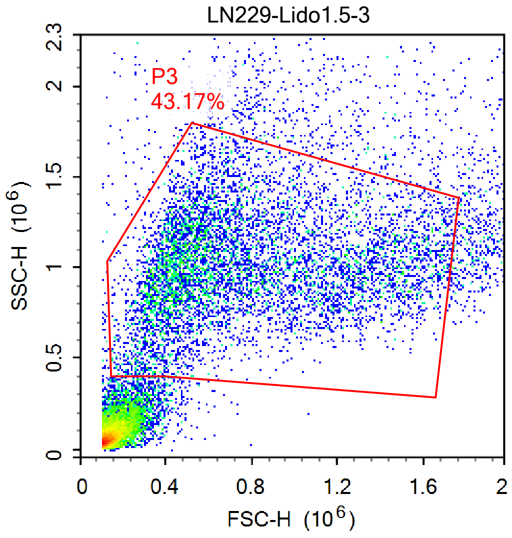

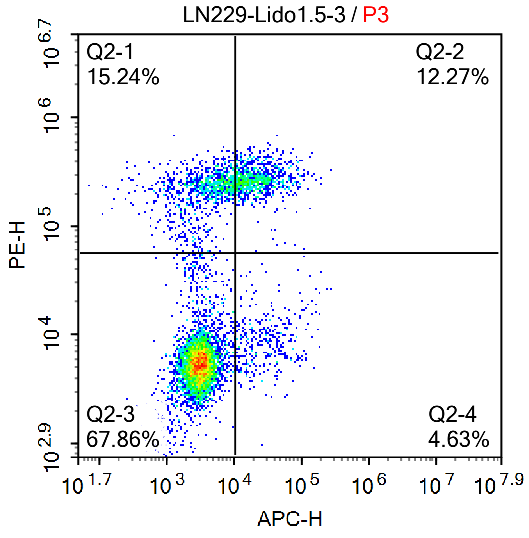

Gate	% All
All	100.00%
P3	43.17%

Gate	% P3	% All
P3	100.00%	43.17%
Q2-1	15.24%	6.58%
Q2-2	12.27%	5.30%
Q2-3	67.86%	29.30%
Q2-4	4.63%	2.00%

样本统计表格 - LN229-Lido1.5-3

Gate	% Parent	% Grandparent	% All
All			
P3	43.17%		43.17%
Q2-1	15.24%	6.58%	6.58%
Q2-2	12.27%	5.30%	5.30%
Q2-3	67.86%	29.30%	29.30%
Q2-4	4.63%	2.00%	2.00%

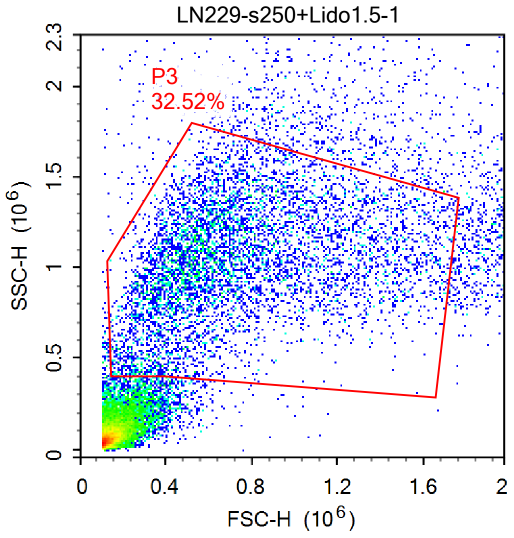

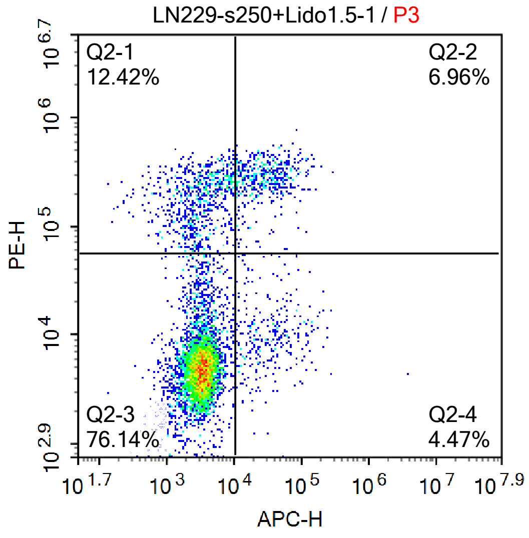

Gate	% All
All	100.00%
P3	32.52%

Gate	% P3	% All
P3	100.00%	32.52%
Q2-1	12.42%	4.04%
Q2-2	6.96%	2.26%
Q2-3	76.14%	24.76%
Q2-4	4.47%	1.45%

样本统计表格 - LN229-s250+Lido1.5-1

Gate	% Parent	% Grandparent	% All
All			
P3	32.52%		32.52%
Q2-1	12.42%	4.04%	4.04%
Q2-2	6.96%	2.26%	2.26%
Q2-3	76.14%	24.76%	24.76%
Q2-4	4.47%	1.45%	1.45%

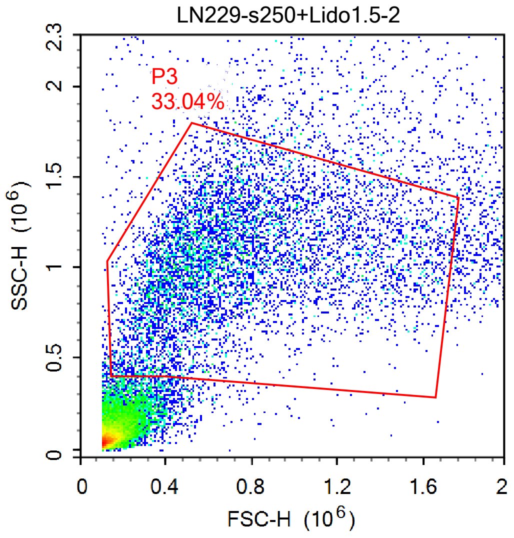

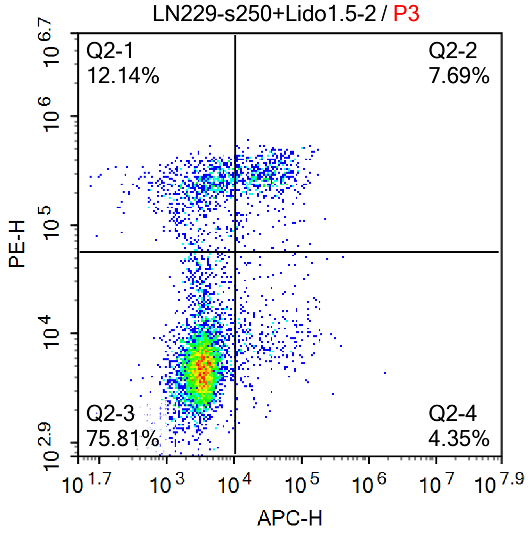

Gate	% All
All	100.00%
P3	33.04%

Gate	% P3	% All
P3	100.00%	33.04%
Q2-1	12.14%	4.01%
Q2-2	7.69%	2.54%
Q2-3	75.81%	25.05%
Q2-4	4.35%	1.44%

样本统计表格 - LN229-s250+Lido1.5-2

Gate	% Parent	% Grandparent	% All
All			
P3	33.04%		33.04%
Q2-1	12.14%	4.01%	4.01%
Q2-2	7.69%	2.54%	2.54%
Q2-3	75.81%	25.05%	25.05%
Q2-4	4.35%	1.44%	1.44%

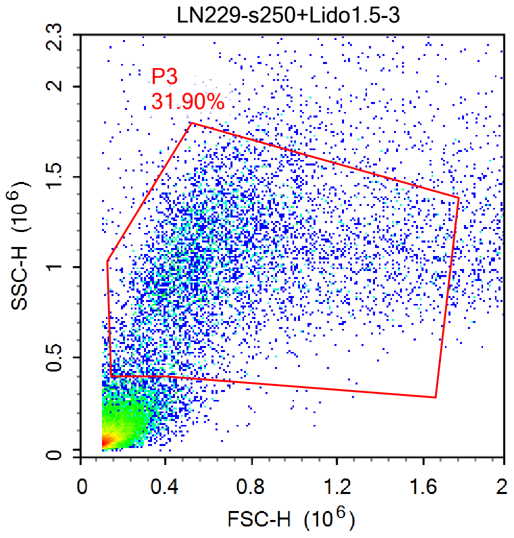

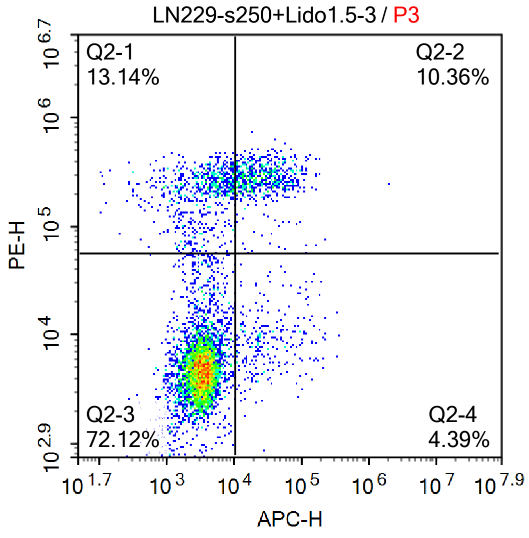

Gate	% All
All	100.00%
P3	31.90%

Gate	% P3	% All
P3	100.00%	31.90%
Q2-1	13.14%	4.19%
Q2-2	10.36%	3.30%
Q2-3	72.12%	23.01%
Q2-4	4.39%	1.40%

样本统计表格 - LN229-s250+Lido1.5-3

Gate	% Parent	% Grandparent	% All
All			
P3	31.90%		31.90%
Q2-1	13.14%	4.19%	4.19%
Q2-2	10.36%	3.30%	3.30%
Q2-3	72.12%	23.01%	23.01%
Q2-4	4.39%	1.40%	1.40%

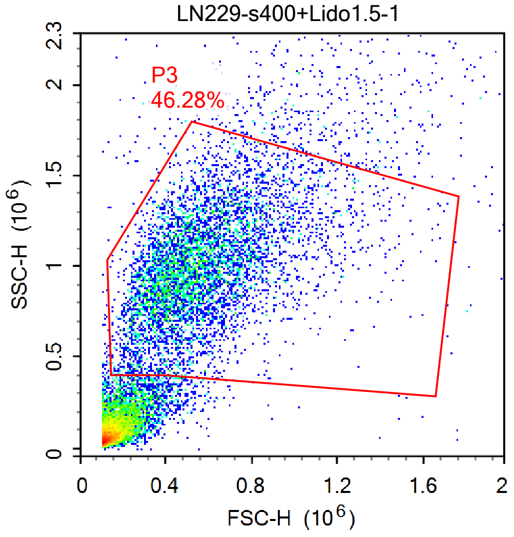

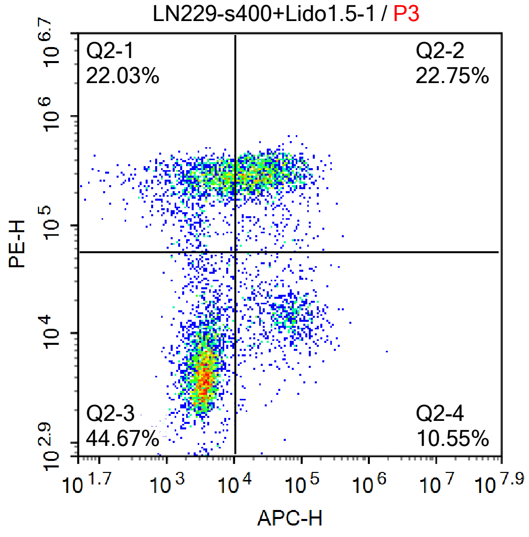

Gate	% All
All	100.00%
P3	46.28%

Gate	% P3	% All
P3	100.00%	46.28%
Q2-1	22.03%	10.20%
Q2-2	22.75%	10.53%
Q2-3	44.67%	20.67%
Q2-4	10.55%	4.88%

样本统计表格 - LN229-s400+Lido1.5-1

Gate	% Parent	% Grandparent	% All
All			
P3	46.28%		46.28%
Q2-1	22.03%	10.20%	10.20%
Q2-2	22.75%	10.53%	10.53%
Q2-3	44.67%	20.67%	20.67%
Q2-4	10.55%	4.88%	4.88%

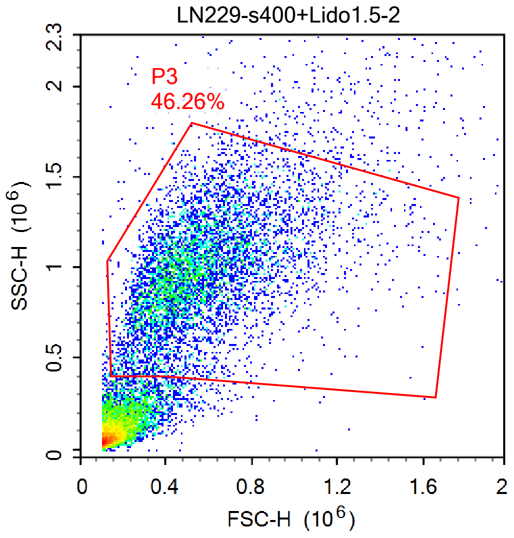

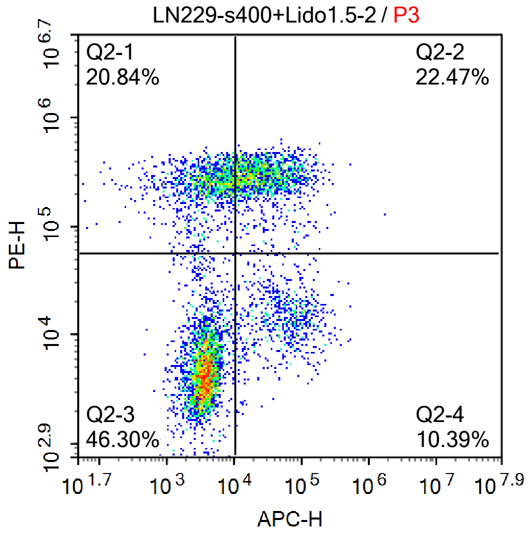

Gate	% All
All	100.00%
P3	46.26%

Gate	% P3	% All
P3	100.00%	46.26%
Q2-1	20.84%	9.64%
Q2-2	22.47%	10.39%
Q2-3	46.30%	21.42%
Q2-4	10.39%	4.81%

样本统计表格 - LN229-s400+Lido1.5-2

Gate	% Parent	% Grandparent	% All
All			
P3	46.26%		46.26%
Q2-1	20.84%	9.64%	9.64%
Q2-2	22.47%	10.39%	10.39%
Q2-3	46.30%	21.42%	21.42%
Q2-4	10.39%	4.81%	4.81%

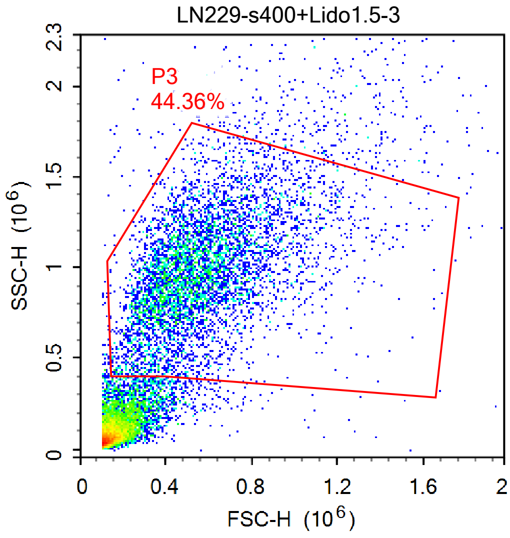

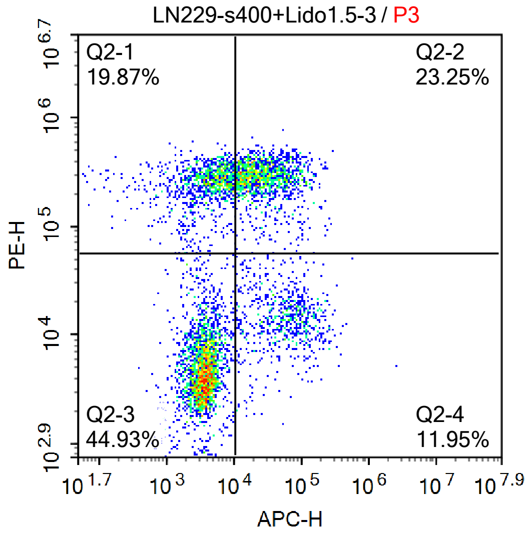

Gate	% All
All	100.00%
P3	44.36%

Gate	% P3	% All
P3	100.00%	44.36%
Q2-1	19.87%	8.82%
Q2-2	23.25%	10.31%
Q2-3	44.93%	19.93%
Q2-4	11.95%	5.30%

样本统计表格 - LN229-s400+Lido1.5-3

Gate	% Parent	% Grandparent	% All
All			
P3	44.36%		44.36%
Q2-1	19.87%	8.82%	8.82%
Q2-2	23.25%	10.31%	10.31%
Q2-3	44.93%	19.93%	19.93%
Q2-4	11.95%	5.30%	5.30%
