# Supplementary material for: Scutellarin combined with lidocaine exerts antineoplastic effect in human glioma associated with repression of epidermal growth factor receptor signaling
Source: PLoS One. 2025 Jan 31;20(1):e0318031. doi: 10.1371/journal.pone.0318031 (PMC11785270; doi:10.1371/journal.pone.0318031)
Supplement: Attachment — Submitted filename: Comments.pdf [file pone.0318031.s019.pdf]

Scutellarin combined with lidocaine attenuates growth of human glioma cells associated with down-regulation of epidermal growth factor receptor

Comments

In the current article, Xiu et al., had found that a combination of Scutellarin and lidocaine attenuates growth of human glioma cells by down-regulation of epidermal growth factor receptor. Overall, the study has several strengths, including the use of multiple assays to assess various aspects of glioma cell behaviour, such as proliferation, migration, and apoptosis. Additionally, investigating the combination of two compounds, scutellarin and lidocaine, adds novelty to the research. However, there are some considerations and areas for improvement in the study design and future research:

Major Suggestions:

- Can you clarify the rationale behind the choice of cell lines used in the study?
- While the general cell culture conditions are provided, it would be beneficial to mention the passage number of the cells used. Continuous passaging can lead to genetic drift and altered cellular behaviour, affecting experimental reproducibility.
- The choice of CCK-8 assay for cell viability analysis is common and suitable. However, the duration of drug intervention (48 hours) seems arbitrary and lacks justification. Providing a rationale for this time frame would enhance the clarity and reproducibility of the experiment.
- I recommend that the authors conduct cytotoxicity analysis on normal astrocytes to confirm the toxic impact of the two compounds.
- Additionally, the effect of these drugs should be compared with the standard drug for glioma viz., Temozolomide through *in vitro* assays to validate their efficacy.
- For the plate clone formation assay, it would be helpful to include information on the criteria used for defining a clone (e.g., minimum number of cells required) and how the counting of clones was performed to ensure consistency and reproducibility.
- Considering the heterogeneity of Glioma, incorporating 3D culture models such as spheroid or organoid cultures would enhance the relevance and translatability of the findings to *in vivo* settings.
- The study proposes a combination therapy approach of Scutellarin and lidocaine against Glioma. Have similar combination therapies been explored in clinical trials or

preclinical studies for GBM, and what are the challenges associated with translating this approach to clinical settings?

- While the study implicates the downregulation of EGFR protein as a potential mechanism for the observed effects, additional experiments has to be conducted to further elucidate the molecular pathways involved. In that case, which downstream signalling cascades will the authors study and why?

Minor Suggestions:

- Check for grammatical accuracy and maintain a consistent writing style.

Overall, the paper has a solid foundation, but refining and expanding on certain aspects will contribute to a more comprehensive and reader-friendly manuscript.
