# Supplementary material for: Scutellarin combined with lidocaine exerts antineoplastic effect in human glioma associated with repression of epidermal growth factor receptor signaling
Source: PLoS One. 2025 Jan 31;20(1):e0318031. doi: 10.1371/journal.pone.0318031 (PMC11785270; doi:10.1371/journal.pone.0318031)
Supplement: Attachment — Submitted filename: Response to Reviewers.docx [file pone.0318031.s020.docx]

**Response to Reviewers**

**Review Comments**

**Reviewer #1:** In this manuscript, the authors tell a story about the effects of Scutellarin-lidocaine on human glioma cells. The results of this work indicated that Scutellarin-lidocaine could repress the proliferation and promote the apoptosis of U251 and LN229 cells. However, there are some problems.

1. Scutellarin and lidocaine have been widely reported in diseases, including various tumors. The authors should cite more references and make more discussions to reveal why such a combination was designed.

**Answer:** Dear reviewer, we appreciate your helpful comment.

As shown in line 562~589 of the revised manuscript, we have made a more detailed discussion on why the combination of scutellarin (SCU) and lidocaine was designed. The details are as follows:

As is known to all, the combination of multi-drugs at low-dose could not only reduce drug resistance and improve effectiveness compared to monotherapy, but also reduce the toxicity of high-dose single drug. Therefore, we studied the combined effect of SCU and lidocaine on glioma. We found that these two drugs held synergistical anti-glioma effect. Actually, studies have reported that SCU and lidocaine not only possessed anti-tumor effect, but also could improve the sensitivity of cancer cells to other chemotherapeutic drugs. For example, SCU could be used as a sensitizer for cisplatin in ovarian cancer, non-small cell lung cancer and prostate cancer, and 5-fluorouracil (5-FU) in colon cancer. Despite being a classic regional anesthetic, lidocaine also enhanced the sensitivity of cisplatin to tumor cells. In hepatocellular carcinoma and breast cancer, combining lidocaine with cisplatin could further suppress tumor development and metastasis compared to cisplatin monochemotherapy. Therefore, co-administration of lidocaine during cisplatin chemotherapy seemed warranted in the clinical treatment of these tumors. In addition to well-known therapeutic agents (cisplatin and 5-FU), a combination with novel anti-tumor agents also induced more potent therapeutic responses. For example, the combination of SCU and C_18_H_17_NO_6_ (caffeoyl tyrosine) further inhibited glioma proliferation/growth and migration and promoted apoptosis compared to single-drug administration. Based on the above evidence, it's not difficult to understand that scutellarin and lidocaine were well-matched with each other to result in more strong anti-glioma responses and lower the toxicity to normal cells.

**2.** Where is SCU from (the manufacturer and product No.), how about its purity? BP? AP? SP? or UP? Would the impurities in the sample affect the cells?

**Answer:** Dear reviewer, thank you for pointing this out.

In this study, scutellarin (CAS No. 27740-01-8) was purchased from MedChemExpress Company (MCE). Its purity has reached 98.56%, which could eliminate the influence of the impurities on cells.

**3.** It's not strange that SCU and lidocaine could affect the proliferation, migration and apoptosis of glioma cells. In many other reports, both SCU and lidocaine could also affect the properties of other tumor cells and normal cells, e.g., in our previous work, SCU and lidocaine could affect vascular endothelial cells, Müller cells and fibroblast cells via regulating STAT3 associated axes. Therefore, my suggestion is to use some normal cells as blank, to investigate their differences with the same drug doses.

**Answer:** Dear reviewer, we humbly acknowledge your invaluable comment.

We agree with you to investigate the effect of SCU and lidocaine on normal cells. In this study, we focused on the toxic effect of SCU and its combination with lidocaine on normal cells. Here, normal astrocytes and cortical neurons from newborn SD rats were isolated, cultured and used for the toxicity analysis. As shown in line 424~454 of the revised manuscript, we found that the toxicity of these two drugs and the combination was very low to astrocytes and neurons.

**4.** Actually, EdU assay is to investigate the DNA synthesis but not proliferation, and PCNA could be used as a proliferation biomarker.

**Answer:** Dear reviewer, many thanks for your comment.

In this study, EdU incorporation assay were performed by Cell-Light EdU Apollo567 *In Vitro* Kit purchased from RiboBio (product code: C10310-1). According to the official directions of this kit, EdU is a thymidine analogue that can replace thymine (T) and infiltrate into DNA molecules being synthesized during DNA replication. Based on the specific reaction between Apollo® fluorescent dye and EdU, DNA replication activity can be directly and accurately detected. Thus, EdU is widely used in research on cell proliferation, differentiation, growth and development, DNA damage repair, etc., especially suitable for cell proliferation screening experiments of compounds and drugs. Therefore, we thought that it was reasonable to use EdU incorporation assay to detect cell proliferation of glioma cells in this study. Of course, we also agree with your suggestion that PCNA could be used as a proliferation biomarker.

**5.** Fig 2 seems confusing, in some cases, low concentration of SCU could even promote the proliferation of U251, and at 400 uM, the addition of lidocaine did not promote the effects of SCU. Therefore, a normal blank cell is necessary here.

**Answer:** Dear reviewer, we humbly acknowledge your invaluable comment.

According to the results from Fig 2, low concentration of SCU (100 or 200 μM) slightly promoted the proliferation of U251 at 24h and 48h (Fig 2. a, b), but held a non-significant inhibitory effect at 72h (Fig 2. c), indicating that the inhibitory effect of SCU might be time-dependent (as shown in line 296-299 and line 311 of the revised manuscript). With regards to why the addition of lidocaine did not promote the effect of SCU at high concentration (400μM), we thought that the reason might be related to the interaction of these two drugs or the capping effect of this combination. This meant that it was better to combine lidocaine with low concentration of SCU, which could not only result in ideal anti-glioma responses, but also reduce the toxicity of these two drugs to normal cells. Of course, we agreed with you that normal blank cells were necessary to be included to investigate the effect of these two drugs. In this study, we focused on the toxic effect of SCU and its combination with lidocaine on normal cells. Here, normal astrocytes and cortical neurons from newborn SD rats were isolated, cultured and used for the toxicity analysis. As shown in Fig 7. c-d, SCU 400μM (instead of 100 and 200 μM) only reduced the cell viability of astrocytes and cortical neurons by 25 %. Moreover, lidocaine 1mM did not intensify the toxicity of SCU on these two normal cells. In a word, the toxicity of these two drugs and the combination was very low to astrocytes and neurons.

**6.** In Figure 4d, the authors should carefully check the apoptosis figures, which reveal similar contours. After scanning by some software, several ones seem like to be the same one at different time.

**Answer:** Dear reviewer, thank for your comment.

We agree with you that based on the apoptosis figures in Fig 4d, it appears that the observed contours are seemingly similar. However, after careful check, these figures are indeed different among the groups. In fact, we only detected the apoptosis of U251 and LN229 cells intervened by SCU and its combination with lidocaine for 48h (the reports for apoptosis analysis by flow cytometry were shown in S1-2 Files). The results in Fig 4d-f came from a single time point (48h), not multiple time points, so no figures in Fig 4d were obtained from different time points within the same group. Of course, in order to improve the reproducibility of the findings, three replicates were set for each group in this experiment, one of which served as the representative figures as shown in Fig 4d.

**7.** The most important problem of this work is the absent of mechanism. The authors indicated that Scutellarin-lidocaine could downregulate EGFR, however, what's the direct target of SCU and what's the target of lidocaine? The section between Scutellarin-lidocaine and EGFR is still a black box. Logically, the authors should at least predict the target of SCU via some online tools, e.g., SwissTargetPrediction, screen some ones and confirm them by Biacore, nano-ITC, IP or in vivo navigation.

In the recent stage, the manuscript is not an integrated story.

**Answer:** Dear reviewer, we humbly acknowledge your invaluable comments.

As you suggested, we firstly predicted the targets of SCU and lidocaine via SwissTargetPrediction online tool (http://www.swisstargetprediction.ch/index.php), a website to estimate the most probable macromolecular targets of a small molecule, assumed as bioactive [1]. According to the predicted results by this online tool (shown in S15 Table), EGFR was indeed the molecular target of SCU, but the interaction between EGFR and lidocaine has not been demonstrated here. However, multiple lines of evidence from research suggested that lidocaine inhibited the progression of lung cancer, colorectal cancer and retinoblastoma via regulating EGFR axis [2-4]. Therefore, we speculated that SCU and even its combination with lidocaine might exert the anti-glioma effect by regulating EGFR signaling. Then, we detected the expression of the key molecule of EGFR signaling in glioma cells - U251and LN229 intervened by SCU and its combination with lidocaine for 48h. Interestingly and dramatically, the results of western blot demonstrated that SCU downregulated the EGFR protein level of both cells in a dose-dependent manner (Fig 8. l, m and S1 Fig). Moreover, the EGFR protein expression of both cells in the lidocaine group was also declined (Fig 8. l, m and S1 Fig). More importantly, the EGFR protein level of U251 and LN229 cells intervened by the combination of SCU and lidocaine was less than that in the groups with SCU or lidocaine alone (Fig 8. l, m and S1 Fig). Based on the above, we concluded that the underlying mechanism that scutellarin combined with lidocaine suppressed the proliferation and migration and induced the apoptosis of glioma cells might be partly associated with the repression of EGFR signaling.

**Reviewer #2:** Scutellarin combined with lidocaine attenuates growth of human glioma cells associated with down-regulation of epidermal growth factor receptor

**Comments**

In the current article, Xiu et al., had found that a combination of Scutellarin and lidocaine attenuates growth of human glioma cells by down-regulation of epidermal growth factor receptor. Overall, the study has several strengths, including the use of multiple assays to assess various aspects of glioma cell ehavior, such as proliferation, migration, and apoptosis. Additionally, investigating the combination of two compounds, scutellarin and lidocaine, adds novelty to the research. However, there are some considerations and areas for improvement in the study design and future research:

**Major Suggestions:**

**1.** Can you clarify the rationale behind the choice of cell lines used in the study?

**Answer:** Dear reviewer, many thanks for your comment.

There are many glioblastoma cell lines available on the website of the American Type Culture Collection (ATCC), including U251, LN229, U87 MG, A172, etc. These cell lines are widely used in the study of gliomas for the following reasons: 1) they can grow stably *in vitro* and are easy to culture and expand; 2) they possess relatively stable genetic characteristics and can maintain genetic stability after multiple passages; 3) they can reflect the characteristics of clinical gliomas, such as cell proliferation, invasiveness, drug sensitivity, etc., providing clinically relevant experimental data for research. Based on this, we indiscriminately included U251 and LN229 cell lines in this study.

**2.** While the general cell culture conditions are provided, it would be beneficial to mention the passage number of the cells used. Continuous passaging can lead to genetic drift and altered cellular behaviour, affecting experimental reproducibility.

**Answer:** Dear reviewer, we appreciate your helpful comment.

As you said, unrestricted continuous passage would lead to genetic drift and alter cellular behaviour, affecting experimental reproducibility. To avoid this, we used U251 and LN229 cell lines with less than 20 passages in this study (see line 118~119 of the revised manuscript). As reported by Gagos, S. et al, despite some karyotypic diversity, colon cancer cell line-SW480 from passage 138 (late passages) and early passages exhibited common marker chromosomes and followed similar patterns of evolution [5]. Based on this evidence, less than 20 passages would not result in changes in the genotype and phenotype of U251 and LN229 cell lines and affect the experimental reproducibility.

**3.** The choice of CCK-8 assay for cell viability analysis is common and suitable. However, the duration of drug intervention (48 hours) seems arbitrary and lacks justification. Providing a rationale for this time frame would enhance the clarity and reproducibility of the experiment.

**Answer:** Dear reviewer, thank you for pointing this out.

According to the results of CCK-8 assay shown in Fig 2a-c, the inhibitory effect of the combination of SCU and lidocaine peaked at 48 h after dosing, so the cell proliferation, apoptosis and migration of U251 and LN229 cells were detected at this time point.

**4.** I recommend that the authors conduct cytotoxicity analysis on normal astrocytes to confirm the toxic impact of the two compounds.

**Answer:** Dear reviewer, we humbly appreciate your suggestion.

We agree with you that the cytotoxicity analysis on normal cells should be conducted to confirm the toxic impact of the two compounds. In this study, normal astrocytes and cortical neurons from newborn SD rats were isolated, cultured and used for the toxicity analysis. As shown in line 424~454 of the revised manuscript and Fig 7, we found that the toxicity of these two drugs and the combination was very low to astrocytes and neurons.

**5.** Additionally, the effect of these drugs should be compared with the standard drug for glioma viz., Temozolomide through in vitro assays to validate their efficacy.

**Answer:** Dear reviewer, many thanks for your suggestion.

The reasons why the standard positive chemotherapeutic drug of glioma, viz. temozolomide, was not included in this study are as follows: 1) glioma cells are usually resistant to temozolomide; 2) the results from our previous study have demonstrated that SCU (200 and 400 μM) harbored a better inhibitory effect on the proliferation and migration of U251 and LN229 cells than temozolomide [6]. Moreover, SCU (200 and 400 μM) also further induced the apoptosis of these two cells [6]. Given that SCU monotherapy was more effective than temozolomide, there was no doubt that the combination also held a better effect.

**6.** For the plate clone formation assay, it would be helpful to include information on the criteria used for defining a clone (e.g., minimum number of cells required) and how the counting of clones was performed to ensure consistency and reproducibility.

**Answer:** Dear reviewer, we acknowledge your comment.

As shown in the figure legend of Fig 2 and the method section of ‘Plate clone formation assay’ (line 161~163 in the revised manuscript), to ensure consistency and reproducibility, the counting of clones was performed by the criteria that one clone has more than 50 cells.

**7.** Considering the heterogeneity of Glioma, incorporating 3D culture models such as spheroid or organoid cultures would enhance the relevance and translatability of the findings to *in vivo* settings.

**Answer:** Dear reviewer, we humbly appreciate your suggestion.

As you suggested, the application of 3D culture models such as spheroid or organoid cultures would enhance the relevance and translatability of the findings to *in vivo* settings. Regretfully, due to limitations in experimental conditions and lack of technology, we are unable to construct these 3D culture models and apologize for this. However, in order to validate the antitumor characteristics of SCU and its combination with lidocaine *in vivo*, we established a xenograft tumor model of glioma and administered these two drugs in nude mice. Our results demonstrated that the tumor size, weight and volume in SCU 50 mg/kg and lidocaine 20 mg/kg groups were significantly smaller than those in control group (Fig 6. a-c and S9 Table), suggesting that SCU and lidocaine with the above doses could effectively inhibit tumor growth *in vivo*. Moreover, the combination achieved a better inhibitory effect on tumor growth (Fig 6. a-c and S9 Table). However, the body weight of nude mice was not obviously different among control, SCU 50 mg/kg, lidocaine 20 mg/kg and the combination groups (Fig 6. d and S9 Table), indicating that the above therapeutic regimens held no effect on the growth of mice. Note: The results and corresponding methods of *in vivo* experiments were shown in line 400~413 and line 212~226 of the revised manuscript, respectively.

**8.** The study proposes a combination therapy approach of Scutellarin and lidocaine against Glioma. Have similar combination therapies been explored in clinical trials or preclinical studies for GBM, and what are the challenges associated with translating this approach to clinical settings?

**Answer:** Dear reviewer, we humbly acknowledge your invaluable comment.

We thought that the challenges associated with translating this approach to clinical settings mainly include the following aspects:

(1) Although SCU and lidocaine have been used in clinical practice and their short-term toxicity has been identified and prevented to an acceptable level, their long-term toxicity to important organs such as heart, brain, liver, lungs, etc. is still unknown.

(2) Despite the aforementioned potent anti-tumor properties of SCU, this compound, like other flavonoids, has poor bioavailability in the intestine due to the lower water solubility, which inevitably hinders its application *in vivo*. Therefore, it is needed to modify its component to obtain more pharmacological activities while retaining its biological properties. It has been shown that SCU can be conjugated to several components (NO, cyclodextrin, long aliphatic chains, and folic acid) in various modifications, leading to the improvement of the pharmacologic activities of this compound [7]. Moreover, conjugation with amide derivates lowered the cytotoxicity of SCU [7], which can be also further achieved with other components and need more investigations.

(3) As a commonly used local anesthetic drug, lidocaine can alter nerve conduction and produce anesthetic effects. When lidocaine is used for anti-tumor treatment, the appropriate dosage should be carefully investigated to avoid anesthesia-like side effects.

(4) Despite that some studies also used animal models, the majority of investigations about the anti-tumor activity of SCU and lidocaine have been conducted *in vitro*. As a result, nothing is known about their anti-tumor activities *in vivo*, particularly in humans. Therefore, it is advised that SCU and lidocaine should be tested in clinical trials to determine whether they may be used in therapeutic regimens and their short-term and long-term toxicity to human body.

**9.** While the study implicates the downregulation of EGFR protein as a potential mechanism for the observed effects, additional experiments has to be conducted to further elucidate the molecular pathways involved. In that case, which downstream signaling cascades will the authors study and why?

**Answer:** Dear reviewer, we humbly acknowledge your invaluable comments.

As mentioned above, to explore the potential mechanism that SCU combined with lidocaine exerted the anti-glioma effect, we firstly predicted the targets of SCU and lidocaine via SwissTargetPrediction online tool (http://www.swisstargetprediction.ch/index.php), a website to estimate the most probable macromolecular targets of a small molecule, assumed as bioactive [1]. According to the predicted results by this online tool (shown in S15 Table), EGFR was indeed the molecular target of SCU, but the interaction between EGFR and lidocaine has not been demonstrated here. However, multiple lines of evidence from research suggested that lidocaine inhibited the progression of lung cancer, colorectal cancer and retinoblastoma via regulating EGFR axis [2-4]. Therefore, we speculated that SCU and even its combination with lidocaine might exert the anti-glioma effect by regulating EGFR signaling. Then, we detected the expression of the key molecule of EGFR signaling in glioma cells - U251and LN229 intervened by these two drugs for 48h. Interestingly and dramatically, the results of western blot demonstrated that SCU downregulated the EGFR protein level of both cells in a dose-dependent manner (Fig 8. l, m and S1 Fig). Moreover, the EGFR protein expression of both cells in the lidocaine group was also declined (Fig 8. l, m and S1 Fig). More importantly, the EGFR protein level in U251 and LN229 cells intervened by the combination of SCU and lidocaine was less than that in the groups with SCU or lidocaine alone (Fig 8. l, m and S1 Fig).

EGFR is known to regulate several downstream signaling pathways such as ERK, PI3K/AKT and JAK/STAT which all play a crucial role in the regulation of cancer stemness, angiogenesis and metastasis [8]. It has been proven that SCU and lidocaine could exert anti-tumor effects by inhibiting these downstream signaling pathways. Firstly, SCU induced cell cycle arrest and apoptosis, and inhibited migration and invasion of lung cancer and human leukemia cells through suppressing ERK, AKT and STAT3 signaling pathways [9-11]. Secondly, lidocaine also blocked tumor progression and recurrence by influencing the activation of ERK and PI3K/AKT signaling pathways [2, 12, 13].

Based on the above, we concluded that the underlying mechanism that SCU combined with lidocaine suppressed the proliferation and migration and induced the apoptosis of glioma cells might be partly associated with the repression of EGFR signaling.

**Minor Suggestions:**

**10.** Check for grammatical accuracy and maintain a consistent writing style.

Overall, the paper has a solid foundation, but refining and expanding on certain aspects will contribute to a more comprehensive and reader-friendly manuscript.

**Answer:** Dear reviewer, many thanks for your comments.

The writing of the manuscript has been revised thoroughly.

**Reviewer #3:** He et al attempted to explore the role of Scutellarin and lidocaine in glioma growth. Both compound inhibited glioma proliferation. They found interesting results. This study open the window for glioma therapeutic approach.

**Answer:** Dear reviewer, many thanks for your comments.

**Note:** All the revisions have been marked with red fonts in the revised manuscript.

**Reference**

1.	Daina A, Michielin O, Zoete V. SwissTargetPrediction: updated data and new features for efficient prediction of protein targets of small molecules. Nucleic acids research. 2019;47(W1):W357-w64.

2.	Sun H, Sun Y. Lidocaine inhibits proliferation and metastasis of lung cancer cell via regulation of miR-539/EGFR axis. Artificial cells, nanomedicine, and biotechnology. 2019;47(1):2866-74.

3.	Xia W, Wang L, Yu D, Mu X, Zhou X. Lidocaine inhibits the progression of retinoblastoma in vitro and in vivo by modulating the miR‑520a‑3p/EGFR axis. Molecular medicine reports. 2019;20(2):1333-42.

4.	Qu X, Yang L, Shi Q, Wang X, Wang D, Wu G. Lidocaine inhibits proliferation and induces apoptosis in colorectal cancer cells by upregulating mir-520a-3p and targeting EGFR. Pathology, research and practice. 2018;214(12):1974-9.

5.	Gagos S, Iliopoulos D, Tseleni-Balafouta S, Agapitos M, Antachopoulos C, Kostakis A, et al. Cell senescence and a mechanism of clonal evolution leading to continuous cell proliferation, loss of heterozygosity, and tumor heterogeneity: studies on two immortal colon cancer cell lines. Cancer genetics and cytogenetics. 1996;90(2):157-65.

6.	He XY, Xu Y, Xia QJ, Zhao XM, Li S, He XQ, et al. Combined Scutellarin and C(18)H(17)NO(6) Imperils the Survival of Glioma: Partly Associated With the Repression of PSEN1/PI3K-AKT Signaling Axis. Frontiers in oncology. 2021;11:663262.

7.	Vesaghhamedani S, Mazloumi Kiapey SS, Gowhari Shabgah A, Amiresmaili S, Jahanara A, Oveisee M, et al. From traditional medicine to modern oncology: Scutellarin, a promising natural compound in cancer treatment. Progress in biophysics and molecular biology. 2023;180-181:19-27.

8.	Talukdar S, Emdad L, Das SK, Fisher PB. EGFR: An essential receptor tyrosine kinase-regulator of cancer stem cells. Advances in cancer research. 2020;147:161-88.

9.	Sun C, Li C, Li X, Zhu Y, Su Z, Wang X, et al. Scutellarin induces apoptosis and autophagy in NSCLC cells through ERK1/2 and AKT Signaling Pathways in vitro and in vivo. Journal of Cancer. 2018;9(18):3247-56.

10.	Bao J, Xia L, Zhao Y, Xia R. Scutellarin exerts anticancer effects on human leukemia cells via induction of Sub-G1 cell cycle arrest, apoptosis and also inhibits migration and invasion by targeting Raf/MEK/ERK signalling pathway. Journal of BUON : official journal of the Balkan Union of Oncology. 2020;25(2):1050-5.

11.	Cao P, Liu B, Du F, Li D, Wang Y, Yan X, et al. Scutellarin suppresses proliferation and promotes apoptosis in A549 lung adenocarcinoma cells via AKT/mTOR/4EBP1 and STAT3 pathways. Thoracic cancer. 2019;10(3):492-500.

12.	Zhang C, Xie C, Lu Y. Local Anesthetic Lidocaine and Cancer: Insight Into Tumor Progression and Recurrence. Frontiers in oncology. 2021;11:669746.

13.	Chen J, Jiao Z, Wang A, Zhong W. Lidocaine inhibits melanoma cell proliferation by regulating ERK phosphorylation. Journal of cellular biochemistry. 2019;120(4):6402-8.
