# Supplementary material for: Scutellarin combined with lidocaine exerts antineoplastic effect in human glioma associated with repression of epidermal growth factor receptor signaling
Source: PLoS One. 2025 Jan 31;20(1):e0318031. doi: 10.1371/journal.pone.0318031 (PMC11785270; doi:10.1371/journal.pone.0318031)
Supplement: Attachment — Submitted filename: Response to Reviewers.docx [file pone.0318031.s021.docx]

**Response to Reviewers**

**Journal Requirements:**

Please review your reference list to ensure that it is complete and correct. If you have cited papers that have been retracted, please include the rationale for doing so in the manuscript text, or remove these references and replace them with relevant current references. Any changes to the reference list should be mentioned in the rebuttal letter that accompanies your revised manuscript. If you need to cite a retracted article, indicate the article’s retracted status in the References list and also include a citation and full reference for the retraction notice.

**Answer:** We have reviewed the reference list and made corrections. Now we ensure that the reference list is complete and correct. And no retracted articles were cited in this manuscript.

**Reviewers' Comments:**

**Reviewer #1**:

1. CCK8 assay is only used to test the DNA synthesis or duplication, but not cell proliferation, it should be clarified in the whole manuscript.

**Answer:** Dear reviewer, many thanks for your comment.

According to the directions, Cell Counting Kit-8 (CCK8) utilizes highly sensitive water-soluble tetrazolium salt-WST-8 as a chromogenic substrate, which can be reduced by intracellular dehydrogenases to produce water-soluble formazan dye. The number of live cells is directly proportional to the amount of formazan dye. Based on this, this kit is usually used for cell counting when detecting the effects of chemical drugs on cell proliferation or toxicity. Therefore, we believe that it is reasonable to use this kit to detect the effect of scutellarin and lidocaine on cell proliferation of glioma cells in this study. In fact, cell proliferation is accompanied by DNA synthesis and replication, so the results of CCK8 assay can also reflect the synthesis and replication of DNA in cells.

2. In the authors' response, "scutellarin (CAS No. 27740-01-8) was purchased from MedChemExpress Company (MCE). Its purity has reached 98.56%, which could eliminate the influence of the impurities on cells." Could a 98.56% purity eliminate the influence of the impurities on cells?

**Answer:** Dear reviewer, thank you for pointing this out.

The description ‘a 98.56% purity eliminate the influence of the impurities on cells’ is incorrect. We apologize for misleading you and the readers with such a description. What we intended to express is that the purity of scutellarin (CAS No. 27740-01-8) is very high, which can reduce the influence of impurities on cells. However, whether or to what extent the impurities make an impact on cells requires further investigation.

3. Could the authors carefully check the apoptosis figures, several sub-images showed the similar contours.

**Answer:** Dear reviewer, we humbly acknowledge your comment.

We agree with you that the observed contours of apoptosis figures from the same cell line in Fig 4d appear similar. However, these figures are portraying different observations from the different interventions experimented in our study. For instance, the figures in the upper row of Fig 4d reflect the apoptosis of U251 cells with different drug interventions, and the figures in the bottom row of Fig 4d show the apoptosis of LN229 cells with different drug interventions. The interventions involved the use of scutellarin drug at different concentrations with or without a 1.5mM lidocaine mixture. These interventions were specifically as follows: the concentration of scutellarin at (i) 250μM without lidocaine, (ii) 400μM without lidocaine, (iii) 250μM with lidocaine, (iv) 400μM with lidocaine, and lastly (v) the cells with only 1.5mM lidocaine. Based on these aforementioned interventions, we can clearly see that both early and late apoptosis in each of the cell lines U251 and LN229, are statistically different. As shown in Fig 4e-f, scutellarin promoted early apoptosis (see the second quadrant (Q4-2) in each single figure of Fig 4d) and late apoptosis (see the fourth quadrant (Q4-4) in each single figure of Fig 4d) of U251 and LN229 cells in a concentration-dependent manner. Lidocaine 1.5 mM also raised the early and late apoptosis of U251 and LN229 cells compared with control group (Fig 4. e, f). What's more, the early and late apoptosis rate of U251 and LN229 cells intervened by the combination of scutellarin and lidocaine 1.5mM was higher than that intervened with scutellarin or lidocaine alone (Fig 4. e, f). We are therefore confident that the figures in Fig 4d are different from each other as they show results of samples taken from different interventions.

The reasons why the observed contours of apoptosis figures appear similar may be as follows: 1) the samples are sourced from the same cell line. As shown in Fig 4d, the figures from the same row appear similar, but the figures from the different row (the upper row: U251 cells; the bottom row: LN229 cells) are obviously different. 2) During the implementation of the experiment, all conditions were controlled uniformly, which reduced the interference of other factors on the experimental results and improved the reproducibility of the findings.

**Reviewer #3**: Combination of Scutellarin and Lidocaine as antineoplastic potential drug for glioma. This is significance study and add new knowledge in glioma tumor therapeutic approach.

**Answer:** Dear reviewer, many thanks for your comments.

**Note:** All the revisions have been marked with red fonts in the revised manuscript.
